# Synovial fluid neutrophils in oligoarticular juvenile idiopathic arthritis have an altered phenotype and impaired effector functions

**DOI:** 10.1186/s13075-021-02483-1

**Published:** 2021-04-09

**Authors:** Sabine Arve-Butler, Tobias Schmidt, Anki Mossberg, Elisabet Berthold, Birgitta Gullstrand, Anders A. Bengtsson, Fredrik Kahn, Robin Kahn

**Affiliations:** 1 Department of Rheumatology, Clinical Sciences Lund, Lund University, Lund, Sweden; 2 Wallenberg Center for Molecular Medicine, Lund University, Lund, Sweden; 3 Department of Pediatrics, Clinical Sciences Lund, Lund University, Lund, Sweden; 4 Department of Infection Medicine, Clinical Sciences Lund, Lund University, Lund, Sweden

**Keywords:** Juvenile idiopathic arthritis, Neutrophil, Synovial fluid, Phenotype, Phagocytosis, Oxidative burst, Reactive oxygen species

## Abstract

**Background:**

Neutrophils are the most prevalent immune cells in the synovial fluid in inflamed joints of children with oligoarticular juvenile idiopathic arthritis (JIA). Despite this, little is known about neutrophil function at the site of inflammation in JIA and how local neutrophils contribute to disease pathogenesis. This study aimed to characterize the phenotype and function of synovial fluid neutrophils in oligoarticular JIA.

**Methods:**

Neutrophils obtained from paired blood and synovial fluid from patients with active oligoarticular JIA were investigated phenotypically (*n* = 17) and functionally (phagocytosis and oxidative burst, *n* = 13) by flow cytometry. In a subset of patients (*n* = 6), blood samples were also obtained during inactive disease at a follow-up visit. The presence of CD206-expressing neutrophils was investigated in synovial biopsies from four patients by immunofluorescence.

**Results:**

Neutrophils in synovial fluid had an activated phenotype, characterized by increased CD66b and CD11b levels, and most neutrophils had a CD16^hi^ CD62L^low^aged phenotype. A large proportion of the synovial fluid neutrophils expressed CD206, a mannose receptor not commonly expressed by neutrophils but by monocytes, macrophages, and dendritic cells. CD206-expressing neutrophils were also found in synovial tissue biopsies. The synovial fluid neutrophil phenotype was not dependent on transmigration alone. Functionally, synovial fluid neutrophils had reduced phagocytic capacity and a trend towards impaired oxidative burst compared to blood neutrophils. In addition, the effector functions of the synovial fluid neutrophils correlated negatively with the proportion of CD206^+^ neutrophils.

**Conclusions:**

Neutrophils in the inflamed joint in oligoarticular JIA were altered, both regarding phenotype and function. Neutrophils in the synovial fluid were activated, had an aged phenotype, had gained monocyte-like features, and had impaired phagocytic capacity. The impairment in phagocytosis and oxidative burst was associated with the phenotype shift. We speculate that these neutrophil alterations might play a role in the sustained joint inflammation seen in JIA.

## Background

Juvenile idiopathic arthritis (JIA) is an inflammatory rheumatic joint disease affecting children. Despite disease onset being at a young age, symptoms may be lifelong and include irreversible joint damage or growth disturbances [1, 2]. The JIA diagnosis is an umbrella term including several subtypes with the common denominator of unexplained persistent arthritis occurring before the age of sixteen [3]. The most common subtype in the Western world is oligoarticular JIA [4], commonly characterized by asymmetric disease onset with inflammation in one to four large joints [3, 5].

Previously, the immunopathogenesis of oligoarticular JIA has been thought to be driven primarily by adaptive immune responses, as the disease is associated with HLA class II genetic variants and the presence of autoantibodies [5]. However, abnormalities in the adaptive immune system cannot fully explain the JIA pathology, and the importance of the innate immune system in oligoarticular JIA is becoming recognized. For instance, gene expression studies in JIA reveal neutrophil activation signatures [6, 7], JIA synovial fluid contains high levels of monocyte-derived cytokines, and synovial monocytes are polarized with impaired phagocytic ability [8, 9]. The importance of the innate immune response is also reflected in the treatment of oligoarticular JIA, where the most commonly used drugs are either non-specific or target important components of the innate immune system such as tumor necrosis factor alpha (TNFα) and interleukin 6 (IL-6), while therapies targeting T and B cells in the adaptive immune system are more seldom used [10, 11].

Neutrophils represent a major part of the innate immune system, and the pathology of several autoimmune rheumatic diseases are at least partly driven by dysfunctional neutrophils [12, 13]. Neutrophils are the most common immune cell present in the synovial fluid from inflamed joints in oligoarticular JIA, and studies of several JIA subtypes suggest that circulating neutrophils are activated [6, 10, 14, 15]. However, neutrophils at the site of inflammation have rarely been studied in JIA, except for a recent study by Metzemaekers et al. which demonstrated that synovial fluid neutrophils in oligo- and polyarticular JIA have a distinct phenotype compared to circulating neutrophils [16].

The major neutrophil effector functions include phagocytosis and oxidative burst. Neutrophil phagocytosis is important not only as a defense against infection but also for clearance of potential autoantigens. Reactive oxygen species (ROS) were long considered harmful byproducts in sterile inflammation but are now recognized for their immunosuppressive properties, and impaired ROS production is associated with rheumatic disease both in patients and animal models of disease [12, 17–23]. Thus far, no studies have investigated synovial fluid neutrophil effector functions in oligoarticular JIA and their relation to the pathogenesis.

The phenotype of synovial fluid neutrophils in JIA is insufficiently characterized, and two contradicting studies describe them as being highly activated [16] or resting [24]. The study by Metzemaekers et al. further describes the synovial fluid neutrophils as hypersegmented and that a significant proportion of the neutrophils expresses markers not usually found on neutrophils, such as HLA-DR [16]. Neutrophils with acquired traits of other myeloid cells, including antigen-presenting capacity and expression of the mannose receptor CD206, have been described in other rheumatic diseases such as rheumatoid arthritis (RA) [25, 26] and adult-onset Still’s disease [27].

In this study, we set out to investigate both phenotype and effector functions of neutrophils in paired blood and synovial fluid from children with oligoarticular JIA. To characterize several important aspects of neutrophil biology, we investigated neutrophil phenotype by surface marker expression related to activation, tissue migration, maturity, and monocyte-like phenotype. Neutrophil function was analyzed in regard to both phagocytosis and ROS production. We hypothesized that synovial fluid neutrophils would differ from circulating neutrophils both regarding phenotype and function and that the neutrophil alterations can be important in driving and maintaining the local joint inflammation in oligoarticular JIA.

## Methods

### Study population and samples

All children fulfilling the International League of Associations for Rheumatology (ILAR) criteria for persistent oligoarticular JIA undergoing therapeutic arthrocentesis between 2016 and 2019 were offered to participate in this study. We included patients who were untreated or solely on non-steroid anti-inflammatory drugs (NSAIDs) for at least 6 months *n* = 17, except for one patient (no. 5) who had received intraarticular steroids in another joint, 2 months prior to sampling. The blood and synovial fluid (from the knee in all patients) during active arthritis were collected from all 17 patients. Synovial tissue biopsies were obtained from four patients, one of which is not included in phenotype or function analyses due to ongoing treatment with methotrexate (patient no. 18), in conjunction with synovial fluid aspiration. Follow-up blood samples from periods of inactive disease, defined as no signs of arthritis and/or uveitis at clinical examination, were collected from six of the patients. During periods of inactive disease, some of the patients were treated with conventional or biologic disease-modifying antirheumatic drugs (DMARD). Patient characteristics are described in Table 1. Twelve of the patients were also included in a previous study [9], indicated in Table 1. Total white blood cell counts and relative frequency of neutrophils were investigated in the blood and synovial fluid samples on an XN-350 instrument (Sysmex Corporation) and can be found in supplemental Table 1.

**Table 1:** Description of patient cohort

Pat#	Sex	Disease duration (months)	Age (years)	Treatment	Uveitis	ANA	Phenotype analysis	Functional assay	Biopsy	Follow-up sample	Time to follow-up (months)	Treatment at follow-up
1*	M	132	15	No	No	Pos	Yes	No	No	No		
2	F	26	7	NSAID	No	Pos	Yes	No	No	No		
3*	F	88	11	No	No	Pos	Yes	Yes	No	Yes	40	No
4*	F	0	6	NSAID	Yes	Pos	Yes	Yes	Yes	No		
5*	F	3	11	NSAID	Yes	Pos	Yes	Yes	No	Yes	34	NSAID
6*	F	49	7.5	NSAID	No	Pos	Yes	No	No	Yes	31	TNFi
7*	F	48	16.5	No	Yes	Pos	Yes	Yes	No	No		
8*	F	2	3.5	No	No	Pos	Yes	Yes	Yes	No		
9*	F	1	15	NSAID	No	Pos	Yes	Yes	No	Yes	20	Mtx, NSAID
10*	M	1	12	NSAID	No	Neg	Yes	Yes	No	No		
11	M	4	10	NSAID	No	Pos	Yes	Yes	No	Yes	19	Mtx
12*	F	0	2	No	No	Pos	Yes	Yes	No	Yes	16	Mtx
13	F	144	17	NSAID	No	Neg	Yes	Yes	No	No		
14*	F	1	13	NSAID	No	Neg	Yes	Yes	No	No		
15	M	2	4	NSAID	No	Pos	Yes	No	No	No		
16	M	0	6	NSAID	No	Pos	Yes	Yes	No	No		
17*	F	1	11	No	No	Neg	Yes	Yes	Yes	No		
18	F	50	5	Mtx	Yes	Pos	No	No	Yes	No		

Clinical and sample data. Disease duration is calculated as months since the date of diagnosis

*Abbreviations*: *TNFi* TNF inhibitor, *Mtx* methotrexate

*Patients are also included in Schmidt et al. [9]

Healthy controls were included after informed consent. All controls were adults and contributed with blood and oral samples.

Blood, synovial fluid, and oral samples were processed and analyzed immediately after collection.

### Preparation of synovial cells and cell-free synovial fluid

Synovial fluid was centrifuged at 400*g*, 10 min to pellet the cells. Synovial cells were counted and resuspended in phosphate-buffered saline (PBS) at a final concentration of 1 × 10^6^/ml for use in flow cytometric staining. The synovial fluid supernatant was transferred to a new tube and centrifuged a second time at 800*g*, 10 min to pellet potential remaining cells or debris. The cell-free synovial fluid was collected and stored at − 80 °C.

### Neutrophil phenotyping and definitions

Equal volumes of blood, synovial cell suspension, or oral cell suspension were stained with antibody panels described in Supplemental Figure 1. Whole blood samples were prepared after staining using TQ-prep with the Immunoprep reagent system (Beckman Coulter) for red blood cell lysis and fixation of white cells. Synovial, oral, and purified cell samples were washed with PBS after staining. Samples were analyzed on a FACS Canto II flow cytometer (BD Biosciences) or Cytoflex (Beckman Coulter) according to Supplemental Figure 1. The Kaluza software (Beckman Coulter) was used for data processing. Gating strategies are described in Supplemental Figure 1.

Neutrophil maturity was determined using surface markers CD62L and CD16, where mature neutrophils are CD16^hi^ and CD62L^hi^, immature neutrophils are CD16^mid^ and CD62L^hi^, and aged neutrophils are CD62L^low^ [28–30]. Activated neutrophils were defined as having high levels of CD11b and CD66b. High levels of CD14 and CD206 were considered features of macrophage/monocyte-like phenotype. Surface marker expression is presented as median fluorescence intensity (MFI) for markers where the population is bell-shaped and the majority of neutrophils have a higher fluorescence than the unstained control (CD16, CD62L, CD10, CD11b, CD66b, and CD14), while presented as percent positive for markers where levels on blood neutrophils are similar to the unstained control (CD206 and S100A8/A9) (see Supplemental Figure 2 for representative histograms for each marker).

### Immunofluorescence staining of synovial tissue biopsies

Paraffin-embedded biopsies in 3-μm sections mounted on microscope glass slides were baked for 1 h at 60 °C followed by deparaffinization in Neo-Clear (Merck) and a series of washes in ethanol of decreasing concentration. Antigen retrieval was performed in citrate buffer pH 6.0. The slides were blocked with 5% bovine serum albumin (BSA) and 5% normal goat serum (Abcam) for 30 min at room temperature prior to staining with primary antibodies rabbit anti-human MPO 1:200 (Dako A0398) and mouse anti-CD206 1:200 (Antibodies-Online clone:22–130) in PBS with 1% BSA at 4 °C overnight. After PBS washes, the slides were stained with secondary antibodies goat anti-rabbit Alexa Fluor 488 (Invitrogen) and donkey anti-mouse Alexa Fluor 568 (Abcam). The slides were mounted with Prolong Gold AntiFade with DAPI (Invitrogen). Images were taken using a Zeiss fluorescence microscope and processed in the Fiji software.

### Stimulation of healthy blood neutrophils with JIA synovial fluid

Neutrophils were isolated from heparinized whole blood from healthy controls using Lymphoprep (Axis-Shield) density gradient centrifugation according to the manufacturer’s instructions followed by sedimentation of red blood cells in saline with 1.5% Dextran T500 (Pharmacosmos). Neutrophils in the supernatant were collected and pelleted, and contaminating red blood cells were lysed with sterile H_2_O. The purified neutrophils, 1 × 10^6^/ml, were incubated in MCDB 131 medium (Gibco) with 20% synovial fluid or 20% serum from a blood donor at 37 °C for 1 h. Following incubation, neutrophils were washed with PBS and phenotypically analyzed by flow cytometry.

### Preparation of neutrophils from the healthy oral cavity

Healthy volunteers performed a mouth rinse with 5 ml 0.9% NaCl for 30 s in the morning, prior to breakfast and toothbrushing. The collected rinse fluid was filtered through 10-μm syringe Filcons (BD Biosciences) and centrifuged at 300*g*, 5 min. The pellet was resuspended in PBS with 0.1% BSA and 2 mM EDTA. Epithelial cells were removed by incubation of biotinylated anti-E-cadherin (Bioss) at a final concentration of 4 μg/ml for 30 min followed by streptavidin-Dynabeads M280 (Invitrogen) and removal by a magnet. The remaining cells were washed with PBS/BSA/EDTA. Leukocytes were isolated using DSB-X™ biotin-labeled anti-CD45 at 2 μg/ml (Dynabeads FlowComp Flexi kit, Invitrogen) and collected with Dynabeads FlowComp™ beads on a magnet. Cells were released from the beads using FlowComp™ release buffer, pelleted, and resuspended in PBS.

### Neutrophil effector functions

Phagocytosis and oxidative burst were analyzed using Phagoburst™ and Phagotest™ assay kit (BD Biosciences) according to the manufacturer’s instructions. Both methods are flow cytometric assays performed on 100 μl heparinized whole blood or synovial fluid, measuring ROS production upon stimulation with phorbol-myristate-acetate (PMA) or opsonized *Escherichia coli* and phagocytosis of fluorescently labeled opsonized *E. coli* respectively*.* When indicated, 90 μl serum or synovial fluid was added to 10 μl whole blood before analysis.

### Statistics

Paired samples were analyzed using Wilcoxon’s matched-pairs signed-rank test. Comparisons between groups of independent data were analyzed using the Mann-Whitney *U* test. Correlations were analyzed using the Pearson *r* correlation coefficient. Statistical analyses were performed in Prism 9.

## Results

### Synovial fluid neutrophils have an aged and activated phenotype

**Fig. 1:**
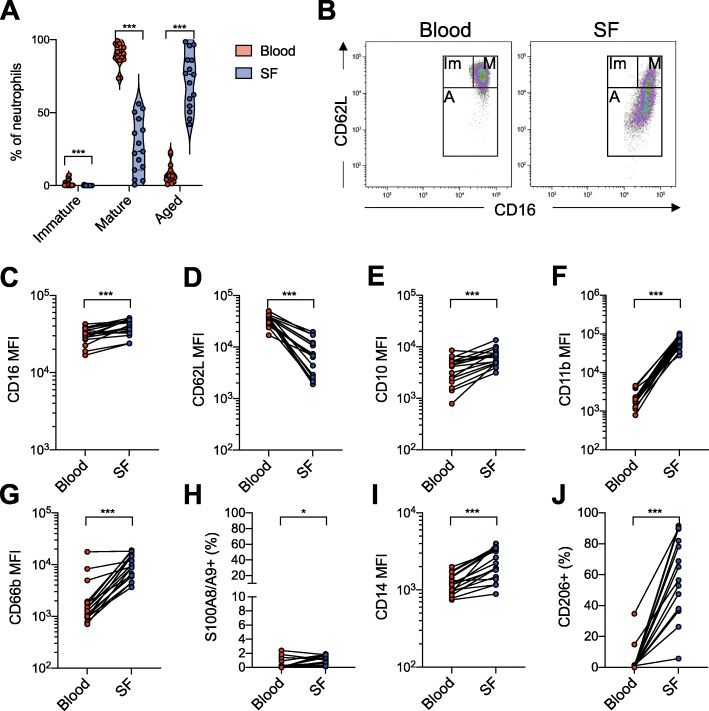
Synovial fluid neutrophils have an aged and activated phenotype and express monocyte-related markers. Neutrophils from paired samples of blood and synovial fluid from patients with oligoarticular JIA were analyzed by flow cytometry. **a** Neutrophil phenotypical maturities in blood and synovial fluid based on CD62L and CD16 expression. **b** Representative plot of neutrophil maturity phenotype gating. Mature neutrophils (M) are CD16^hi^ CD62L^hi^, immature neutrophils (Im) are CD16^mid^ CD62L^hi^, and aged A neutrophils are CD62L^low^. **c**–**g**, **i** Median fluorescence intensity (MFI) of neutrophil expression of **c** CD16, **d** CD62L, **e** CD10, **f** CD11b, **g** CD66b, and **i** CD14. **h**, **j** Proportion of neutrophils, presented as % of total neutrophils, with cell surface expression of **h** S100A8/A9 and **j** CD206. *p < 0.05, ***p < 0.001, Wilcoxon signed-rank test

Blood neutrophils in all patients were primarily of a mature phenotype (CD16^hi^, CD62L^hi^), with very few immature (CD16^mid^, CD62L^hi^) or aged (CD62L^low^) neutrophils. However, in the synovial fluid, most neutrophils had an aged phenotype (range 41–99%, median 76%), and no immature neutrophils were observed (Fig. 1a–d). Synovial fluid neutrophils also had higher levels of the age-related marker CD10 compared to neutrophils in the circulation (Fig. 1e).

Neutrophils in the synovial fluid had an activated phenotype, demonstrated by increased levels of activation markers CD11b and CD66b (Fig. 1f, g), and low levels of CD62L, which is shed upon neutrophil activation and/or migration. Despite the activated phenotype, S100A8/A9, a protein released from activated neutrophils, was only present on the surface of less than 3% of both synovial fluid and blood neutrophils (Fig. 1h).

### Synovial fluid neutrophils express monocyte/macrophage-related surface markers

Synovial fluid neutrophils had increased CD14 levels compared to the circulating neutrophils (Fig. 1i). Mannose receptor CD206, not commonly known as a neutrophil receptor, but expressed by monocytes, macrophages, and dendritic cells, was found on a significant proportion of synovial fluid neutrophils (range 6–92%, median 56%), while being low or non-existent on circulating neutrophils (Fig. 1j). The increased expression of both CD14 and CD206 might suggest that the synovial fluid neutrophils gain a monocyte/macrophage-like phenotype.

### CD206-expressing neutrophils are found in tissue biopsies

**Fig. 2:**
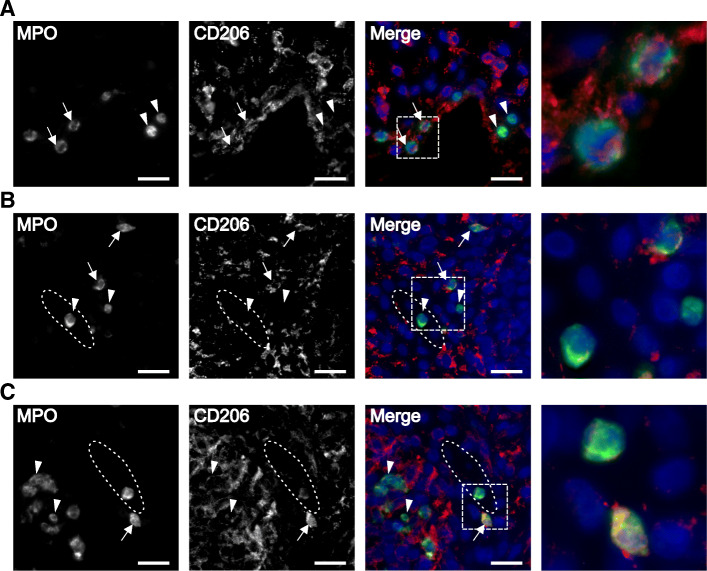
CD206-expressing neutrophils are found in synovial tissue. Synovial tissue biopsies from three patients, stained for MPO (green), CD206 (red), and DAPI (blue). Representative images of **a** patient 4, **b** patient 8, and **c** patient 18. Neutrophils expressing CD206, indicated with arrows, and neutrophils without CD206 expression, indicated with arrow heads, were found in all three biopsies. The synovial vessels are indicated with dotted ellipses. Neutrophils were found both scattered in the synovial tissue and inside the synovial blood vessels. The fourth image in each panel represents a magnification of the area indicated in the merged image. Scale bar is 20 μm. All images are taken at × 40 magnification

To confirm the finding of CD206^+^ neutrophils in the synovial fluid, we investigated whether neutrophils migrating through synovial tissue express CD206. Synovial tissue biopsies obtained from four patients were stained for CD206 and myeloperoxidase (MPO). Neutrophils co-expressing both MPO and CD206 were observed in tissue biopsies from patients 4, 8, and 18 (Fig. 2, arrows), demonstrating that CD206-expressing neutrophils can be present in synovial tissue. In all biopsies, there were also neutrophils without CD206 expression (Fig. 2, arrowheads). In two of the biopsies, we observed synovial blood vessels, and the circulating neutrophils within the vessels had no or low CD206 expression (Fig. 2b, c). The biopsy from patient 17 did not contain any area with neutrophils (data not shown).

### Circulating neutrophils are similar during inactive disease and flares

**Fig. 3:**
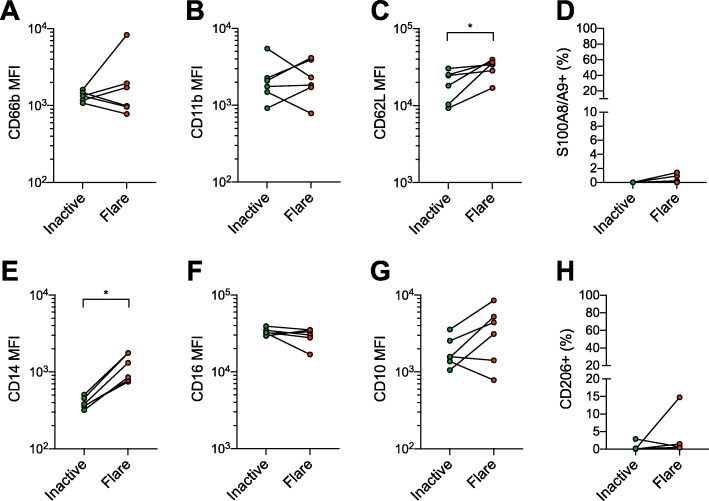
Neutrophil phenotypes in JIA blood are similar during flare and inactive disease. Neutrophil surface marker levels in paired blood samples taken during arthritis flare or inactive disease without arthritis. **a**–**c**, **e**–**g** Median fluorescence intensity (MFI) of neutrophil expression of **a** CD66b, **b** CD11b, c CD62L, **e** CD14, **f** CD16, and **g** CD10. **d**, **h** Proportion of neutrophils, presented as % of total neutrophils, with cell surface expression of **d** S100A8/A9 and **h** CD206. *p < 0.05, Wilcoxon signed-rank test

Patients included in the study did not exhibit systemic symptoms. Previous literature however suggests that circulating neutrophils in JIA are activated during flares [6, 14, 31]. We therfore compared blood neutrophil phenotypes during flare and inactive disease to investigate potential systemic neutrophil alterations. We did not observe any systemic activation of neutrophils during flare; neutrophil activation markers CD66b and CD11b were mostly unchanged, CD62L levels slightly increased, and cell surface S100A8/A9 was present on less than 3% of the neutrophils (Fig. 3a–d). CD14 levels were markedly increased during flare (Fig. 3e), while levels of CD16 and CD10 were not significantly altered (Fig. 3f, g). CD206 expression, which was low or non-existent on blood neutrophils during flare, was equally low during inactive disease (Fig. 3h). Together, these results indicate that neutrophils are relatively unchanged systemically during flares, while being significantly altered at the site of inflammation.

### The synovial neutrophil phenotype is not dependent on transmigration or synovial fluid alone

**Fig. 4:**
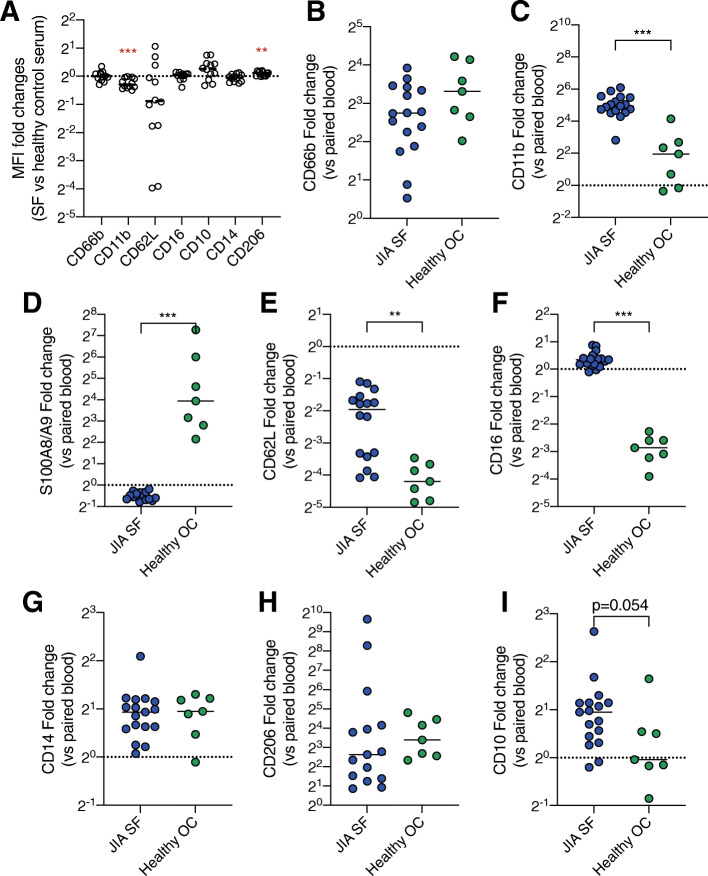
The neutrophil phenotype shift is not explained by exposure to synovial fluid or transmigration. **a** Healthy control blood neutrophils were challenged with 20% JIA synovial fluid (*n* = 12) or 20% serum from the healthy blood donor for 1 h followed by the analysis of surface marker expression. Control serum-stimulated neutrophils are represented by the dotted line. Data are presented as fold change of median fluorescence intensity (MFI) in synovial fluid stimulated compared to control neutrophils. **b**–**i** Neutrophils in JIA synovial fluid (SF) were compared to neutrophils from the oral cavity (OC) of healthy controls, to investigate the alterations in surface markers between the blood and tissue in paired samples of blood and SF or blood and OC. Blood values are represented by the dotted line. Data are presented as fold change of MFI values of **b** CD66b, **c** CD11b, **d** S100A8/A9, **e** CD62L, **f** CD16, **g** CD14, **h** CD206, and **i** CD10 in tissue neutrophils compared to paired blood neutrophils. Line at the median. **p < 0.01, ***p < 0.001, Mann-Whitney *U* test

To investigate if the synovial fluid neutrophil phenotype found in the JIA patients could be explained by exposure to synovial fluid, healthy blood neutrophils were treated with synovial fluid in vitro. Exposure to synovial fluid did not induce any major phenotype shift on healthy blood neutrophils (Fig. 4a). Most surface markers remained unchanged except CD11b, which, opposite to the findings in patients, was significantly lower after exposure to synovial fluid. There was a very modest increase of CD206 (Fig. 4a). These results suggest that the phenotype shift between circulating and synovial fluid neutrophils cannot be explained by exposure to synovial fluid alone.

To investigate if tissue transmigration towards other sites would result in a phenotype shift similar to synovial fluid neutrophils, we investigated transmigrated neutrophils from the oral cavity of healthy controls. The phenotype shifts between healthy blood and oral cavity neutrophils were compared with JIA blood and synovial fluid neutrophils. Neutrophils in both the oral cavity and joint were activated compared to their blood counterparts (Fig. 4b, c). CD11b surface expression was elevated in the transmigrated neutrophils at both sites but more pronounced in the synovial fluid compared to oral neutrophils (Fig. 4c). On the other hand, neutrophil surface S100A8/A9 was markedly increased on oral cavity neutrophils but not on synovial fluid neutrophils (Fig. 4d). The levels of CD62L were lower in oral neutrophils than in synovial fluid neutrophils, compared to their circulating counterparts (Fig. 4e). Oral neutrophils had dramatically decreased levels of CD16 while synovial fluid neutrophils had a small increase in this marker (Fig. 4f). Both oral and synovial fluid neutrophils had increased levels of CD14 and CD206 (Fig. 4g, h). CD10 levels were more increased in synovial compared to oral neutrophils, despite not reaching statistical significance (Fig. 4i). Taken together, transmigration towards both sites induced a shift in neutrophil phenotypes, but with distinctly different surface marker patterns.

### Synovial fluid neutrophils have impaired phagocytosis 

**Fig. 5:**
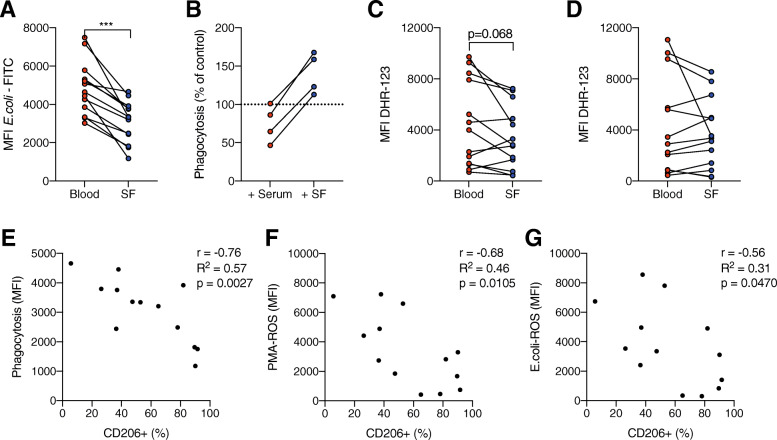
Neutrophil effector functions are impaired in JIA blood and synovial fluid. **a** Phagocytosis of fluorescently labeled opsonized *E. coli* in neutrophils from paired samples of JIA blood and synovial fluid (SF). **b** Phagocytosis of fluorescently labeled opsonized *E. coli* in healthy control neutrophils in a mixture of 10% whole blood and 90% serum or cell-free synovial fluid from four JIA patients. The results are presented as phagocytosis, measured as MFI, in serum/SF-treated samples compared to non-treated whole blood. **c**, **d** Neutrophil ROS production, quantified using DHR-123 fluorescence, after stimulation with **c** PMA or **d** opsonized *E. coli*. **e**–**g** Pearson *r* correlation analysis of the proportion of CD206^+^ neutrophils in the synovial fluid with **e** phagocytosis of opsonized *E. coli*, **f** ROS production by PMA-stimulation, and **g** ROS production by opsonized *E. coli* stimulation. ***p < 0.001, Wilcoxon signed-rank test. MFI, median fluorescence intensity

Neutrophil effector functions phagocytosis and oxidative burst were evaluated in blood and synovial fluid. Synovial fluid neutrophils had a significantly decreased ability to phagocytose opsonized *E. coli* compared to circulating neutrophils (Fig. 5a). The impaired phagocytosis was not due to presence of synovial fluid in the assay, as dilution of healthy control blood in cell-free JIA synovial fluid led to an increase in neutrophil phagocytic ability, in contrast to dilution in JIA serum which led to a slight impairment in phagocytosis (Fig. 5b). Upon stimulation with PMA, there was a trend towards impaired ROS production in synovial fluid neutrophils compared to circulating neutrophils, although not reaching statistical significance (Fig. 5c). ROS production upon stimulation with opsonized *E. coli* was similar in synovial and circulating neutrophils (Fig. 5d). Neutrophil capacity of phagocytosis and oxidative burst was not influenced by patient age (Supplemental Figure 3).

### The proportion of CD206^+^ neutrophils correlates with impaired phagocytosis and oxidative burst

Monocytes typically have a lower capacity for both phagocytosis and oxidative burst compared to neutrophils, and we therefore hypothesized that CD206 expression on synovial fluid neutrophils might be associated with altered effector functions. Indeed, we observed a negative correlation of the proportion of CD206^+^ neutrophils with both phagocytosis of opsonized *E. coli* (Fig. 5e) and ROS production (Fig. 5f, g) in synovial fluid neutrophils, supporting the hypothesis that synovial fluid neutrophils have attained a more monocyte-like phenotype.

## Discussion

In this study we show that synovial fluid neutrophils in active oligoarticular JIA have an activated, aged, and transmigrated phenotype. The neutrophils also have monocyte-like surface marker expression both in the synovial fluid and when migrating within the synovial membrane. This monocyte-like phenotype is associated with reduced phagocytic capacity and ROS production. Thus, we suggest that neutrophils with altered functions are driving or sustaining the local inflammation in the joints of children with oligoarticular JIA.

In a healthy joint, the synovial fluid normally contains no or few immune cells. During arthritis, immune cells are recruited from the bloodstream by chemotactic stimuli. Neutrophils found in the synovial fluid have extravasated from the synovial blood vessels and migrated through the synovial tissue that contains both synovial fibroblasts and infiltrating immune cells. In our study, the majority of these transmigrated neutrophils in the synovial fluid had an aged phenotype, characterized by low surface levels of l-selectin (CD62L) and increased CD10 compared to neutrophils in the blood. The high amount of neutrophils with an aged phenotype in the joint could be due to the anti-apoptotic effects of synovial fluid and inflammatory stimulation [32, 33]. The presence of neutrophils with an aged phenotype in the synovial fluid is supported by a recent study on synovial fluid neutrophils in oligo- and polyarticular JIA, which described the majority of synovial fluid neutrophils to be of a CD62L^low^ and hypersegmented phenotype [16]. However, CD62L is also shed upon neutrophil activation and migration [34]. Thus, the CD62L^low^ neutrophils seen in the synovial fluid could be a consequence of both aging and/or activation and tissue migration. Neutrophils may become activated by a wide variety of stimuli, such as inflammatory cytokines and chemotactic stimulation [35], and activated neutrophils present at a site of inflammation are expected. Indeed, synovial fluid neutrophils were highly activated, measured as increased levels of CD11b and CD66b. Nevertheless, neutrophils in the non-inflammatory oral cavity also had an increase in CD11b and CD66b, as these adhesion molecules are increased upon tissue migration. The activated phenotype of synovial fluid neutrophils is probably a consequence of combined inflammatory stimulation and tissue migration. In contrast, circulating neutrophils are rather unaffected by disease flares, demonstrated by the very similar levels of neutrophil surface markers during active and inactive disease. These findings are in line with the clinical presentation of the disease, where patients with oligoarticular JIA rarely experience systemic symptoms.

A large proportion of the synovial fluid neutrophils expressed the mannose receptor CD206, a receptor usually expressed by monocytes, macrophages, and dendritic cells, which is upregulated on synovial fluid monocytes in JIA [9]. In macrophages, increased expression of CD206 is associated with the “M2” phenotype and anti-inflammatory functions [36], but the role of CD206 on neutrophils is unknown. To our knowledge, only one study has described CD206 on human neutrophils; in this study of adult-onset Still’s disease, the authors found that CD206 appeared on circulating neutrophils during flare but not during inactive disease [27]. CD206^+^ neutrophils are also described in a few publications of mouse models of stroke and myocardial infarction, where they are called “N2” neutrophils in analogy with M2 macrophages [37–39]. In one of these studies, CD206 could be induced on murine neutrophils by in vitro IL-4, a cytokine known to induce CD206 expression on monocytes and macrophages [39, 40]. We confirmed the presence of CD206^+^ neutrophils in synovial biopsies where we found that some, but not all, of the neutrophils scattered within the synovial tissue had expression of CD206. The CD206 mannose receptor recognizes mannosylated and sulfated sugars and has a wide variety of functions including phagocytosis and promotion of antigen presentation [41].

It seems as if the synovial fluid neutrophils are gaining a phenotype with features of both the neutrophil and monocyte lineages, based on the large proportion of CD206^+^ neutrophils and increased levels of the TLR4 co-receptor CD14. Neutrophils expressing surface markers related to other myeloid phenotypes have previously been found in the synovial fluid in JIA and RA. In the recent study of JIA synovial fluid neutrophils by Metzemaekers et al., a significant portion of the synovial fluid neutrophils had gained expression of HLA-DR [16], and synovial fluid neutrophils from RA patients have also been found to have antigen-presenting abilities [25, 26]. As CD206 is described to be involved in the cross-presentation of antigens [41], it is possible that expression of this receptor mediates antigen-presenting capacity of the neutrophils. Neutrophil plasticity to transdifferentiate into cells of other myeloid lineages is well established in vitro [42–44], and we believe that the microenvironment in the inflamed joint is affecting neutrophils to acquire other myeloid traits.

The phenotype shift seen in synovial fluid neutrophils is not dependent on either synovial fluid or transmigration separately. In vitro exposure of JIA synovial fluid to healthy blood neutrophils did not cause a phenotype shift. We further studied oral cavity neutrophils as a control for tissue migration towards a non-inflammatory site. The synovial fluid and oral cavity are very different, where the oral cavity for instance carries a microflora while the synovial fluid is sterile, but still, we found that neutrophils at both sites shared some common features (activation, loss of CD62L, and gain of CD14 and CD206). However, the extent of the alterations was different in the two locations, and other changes occurred in only one of the sites (increase of CD10 in synovial fluid, increase of S100A8/A9 in the oral cavity, and decrease of CD16 in oral cavity). The findings of distinct phenotypes varying between the synovial fluid and oral cavity are supported by previous publications studying neutrophils in the synovial fluid compared to skin blister fluid or pleural effusions [16, 24]. Our results indicate that the synovial neutrophil phenotype and function alterations are induced by a combination of multiple factors and stimuli, and further studies are needed to characterize this.

Importantly, we found a significant decrease in phagocytic capacity of the synovial fluid neutrophils compared to circulating neutrophils. An impaired neutrophil function may drive inflammation and autoimmunity, as neutrophil clearance of debris and dead cell remnants is essential to maintain homeostasis and minimize the autoantigenic burden [12]. Extracellular ROS produced by neutrophils are also important for immunoregulation [20, 45], and impaired neutrophil ROS production in the joint could prolong the resolution of inflammation. As we demonstrate that the addition of synovial fluid to healthy control neutrophils does not impair phagocytic capacity, we suggest that the impairment in effector functions is an inherent trait of the synovial fluid neutrophils and not a consequence of the local microenvironment.

Neutrophil activation or priming is often associated with increased effector functions [35], while our results indicate an opposite relation with an activated phenotype and impaired effector functions. One explanation for this might be that the neutrophils in the synovial fluid are in a state of “exhaustion,” where they have already executed their effector functions and are unresponsive to further stimulation. This phenomenon is common in sepsis and sometimes called immunoparalysis [46]. In addition, as the reduced capacity of phagocytosis and oxidative burst was associated with the phenotype shift of synovial neutrophils towards a monocyte-like pattern with increased surface expression of CD206, we speculate that alterations in phenotype and function could be connected. Indeed, monocytes and macrophages typically have a lower capacity for both phagocytosis and oxidative burst in our functional assay, and the association between impaired neutrophil function and increased proportion of CD206^+^ neutrophils might indicate that the neutrophils are becoming monocyte-like in regard to both phenotype and function. We speculate that the shift in neutrophil phenotype and function between the blood and synovial fluid in oligoarticular JIA could be an interesting target for the development of future therapies.

The neutrophil gating strategy for patient neutrophils and the fact that more aspects of synovial fluid neutrophil effector functions were not investigated present some limitations to the study. The panels were designed for negative selection of neutrophils, but as synovial fluid may contain cells and particles with neutrophil size and granularity without leukocyte surface markers, a positive selection marker was also needed. After negative selection, neutrophils were therefore selected by CD16 positivity, which may inadvertently lose immature CD16^low^ neutrophils. Neutrophil function is not limited to oxidative burst and phagocytosis, and to further characterize the functional differences between circulating and synovial fluid neutrophils in JIA including chemotaxis, the release of neutrophil extracellular traps (NETs) and acquired ability to present antigens would be of interest and importance. These possible functional alterations will be investigated in further studies.

## Conclusions

This study demonstrates that neutrophils in the synovial fluid of children with active oligoarticular JIA are altered both phenotypically and functionally compared to paired neutrophils from the circulation. We observed that synovial fluid neutrophils are activated, aged, and has gained a monocyte-like phenotype, which is associated with an impairment in neutrophil phagocytosis and oxidative burst. We suggest that these neutrophil alterations could be of importance in sustaining joint inflammation and thus interesting possible targets for specific therapies.

## Supplementary Information

**Additional file 1 **: **Supplemental table I.** Absolute leukocyte counts and relative neutrophil frequencies in blood and synovial fluid.

**Additional file 2 **: **Supplemental Figure 1.** Neutrophil gating strategies. **Supplemental Figure 2.** Neutrophil surface markers. **Supplemental Figure 3.** Patient age does not influence neutrophil phagocytosis or oxidative burst.

## Data Availability

Anonymized datasets used and/or analyzed during the current study are available from the corresponding author on reasonable request.

## Abbreviations

JIA: Juvenile idiopathic arthritis
ROS: Reactive oxygen species
PMA: Phorbol 12-myristate 13-acetate
NSAID: Non-steroid anti-inflammatory drug
MFI: Median fluorescence intensity
RA: Rheumatoid arthritis
