# Supplementary material for: Synovial fluid neutrophils in oligoarticular juvenile idiopathic arthritis have an altered phenotype and impaired effector functions
Source: Arthritis Res Ther. 2021 Apr 9;23:109. doi: 10.1186/s13075-021-02483-1 (PMC8034063; doi:10.1186/s13075-021-02483-1)
Supplement: Supplementary file 1 — Additional file 1 : Supplemental table I. Absolute leukocyte counts and relative neutrophil frequencies in blood and synovial fluid. [file 13075_2021_2483_MOESM1_ESM.docx]

| **Pat#** | **WBC blood** | **Neut (%) blood** | **WBC SF** | **Neut (%) SF** | **CD206+ (% of SF neut)** | **WBC blood follow-up** | **Neut (%)**  **blood follow-up** |
| --- | --- | --- | --- | --- | --- | --- | --- |
| 1 | 6.5 | - | - | - | - |  |  |
| 2 | - | - | - | - | - |  |  |
| 3 | 4.9 | - | - | - | 36.4 | 5.97 | 61.0 |
| 4 | 8.2 | - | - | - | 78.9 |  |  |
| 5 | 6.1 | 60.6 | - | - | 91.6 | 6.26 | 74.7 |
| 6 | - | - | - | - | 56.4 | 7.2 | - |
| 7 | 4.6 | 58.7 | 1.7 | 28.5 | 89.6 |  |  |
| 8 | 7.1 | 51.6 | 1.1 | * | 5.7 |  |  |
| 9 | 8.4 | 53.8 | 2.4 | 11.9 | 37.1 | 4.28 | 54 |
| 10 | 8.3 | 71.0 | 5.8 | 85.5 | 47.5 |  |  |
| 11 | 6.1 | 43.7 | 9.2 | 39.9 | 65.0 | 4.61 | 45.1 |
| 12 | 11.6 | 38.6 | 4.7 | 27.9 | 26.3 | 8.44 | 40.7 |
| 13 | 7.0 | 69.3 | 2.7 | 56.3 | 52.9 |  |  |
| 14 | 5.8 | 56.1 | 1.9 | 28.6 | 38.0 |  |  |
| 15 | 4.7 | 53.2 | 4.5 | 71.0 | 68.8 |  |  |
| 16 | 6.2 | 47.2 | 5.8 | 40.4 | 82.0 |  |  |
| 17 | 2.7 | 55.9 | 0.5 | * | 90.1 |  |  |

**Supplemental table I.** Absolute leukocyte counts and relative neutrophil frequencies in blood and synovial fluid. Abbreviations: WBC = white blood cell count (x10^6^/ml), Neut = neutrophils (% of total WBC), SF = synovial fluid. Symbols: ”-” = missing data, ”*” = too few cells to obtain differential data.
