# Supplementary material for: Synovial fluid neutrophils in oligoarticular juvenile idiopathic arthritis have an altered phenotype and impaired effector functions
Source: Arthritis Res Ther. 2021 Apr 9;23:109. doi: 10.1186/s13075-021-02483-1 (PMC8034063; doi:10.1186/s13075-021-02483-1)
Supplement: Supplementary file 2 — Additional file 2 : Supplemental Figure 1. Neutrophil gating strategies. Supplemental Figure 2. Neutrophil surface markers. Supplemental Figure 3. Patient age does not influence neutrophil phagocytosis or oxidative burst. [file 13075_2021_2483_MOESM2_ESM.pdf]

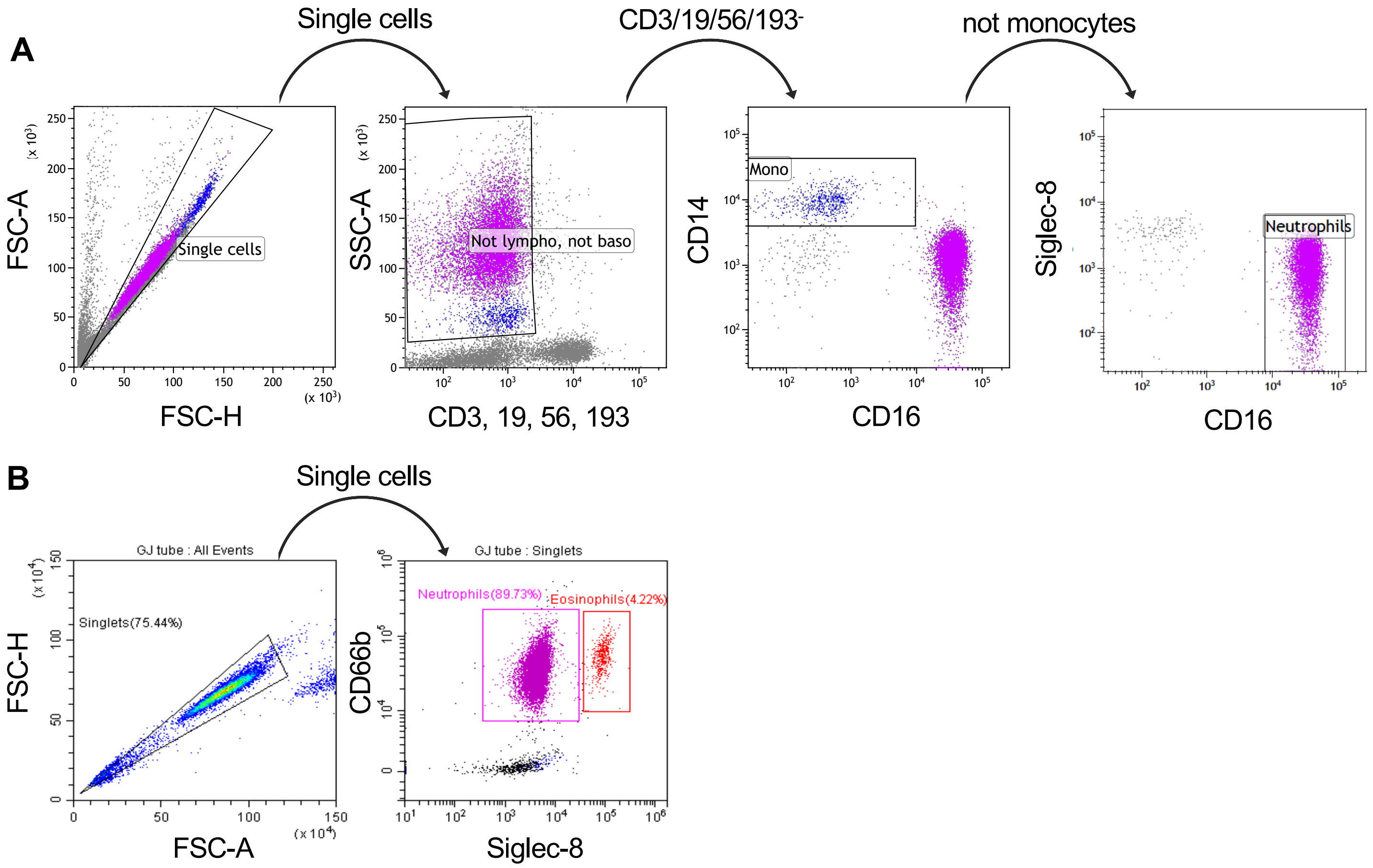

C

1	2	3
CD3-BV510 (BD)	-”-	-”-
CD19-BV510 (BD)	-”-	-”-
CD193-BV510 (BD)	-”-	-”-
CD56-BV510 (BD)	-”-	-”-
CD14-BV421 (Biolegend)	-”-	-”-
CD16-APCH7 (BD)	-”-	-”-
Siglec-8-PE (Biolegend)	-”-	-”-
CD10-PECy7 (BD)		
CD11b-APC (BD)	CD206-APC (BD)	CD62L-APC (eBiosciences)
	CD66b-FITC (BD)	S100A8/A9-FITC (Santa Cruz)

4
CD66b-FITC (BD)
Siglec-8-BV510 (BD)
CD11b-AF700 (BD)
CD16-APCH7 (BD)
CD14-BV421 (Biolegend)
CD206-PE (BD)
CD10-PECy7 (BD)
CD62L-APC (eBiosciences)

Supplemental Figure 1. Neutrophil gating strategies.
A) Gating strategy for neutrophils in blood, synovial fluid and oral cavity samples. Single cell selection followed by negative selection of lymphocyte and basophil markers CD3, CD19, CD56 and CD193. Monocytes were identified as CD14^{high}, CD16$^{mid/low}$. Neutrophils were separated from eosinophils by CD16 and low/intermediate Siglec-8. B) Gating strategy for purified neutrophils after *in vitro* exposure to synovial fluid. Single cell selection followed by identification as CD66b^{high}, Siglec-8^{mid}. C) Antibody panels used for samples in A (panel 1-3) and B (panel 4). Boxes marked –”- indicates the same antibody as used in panel 1.

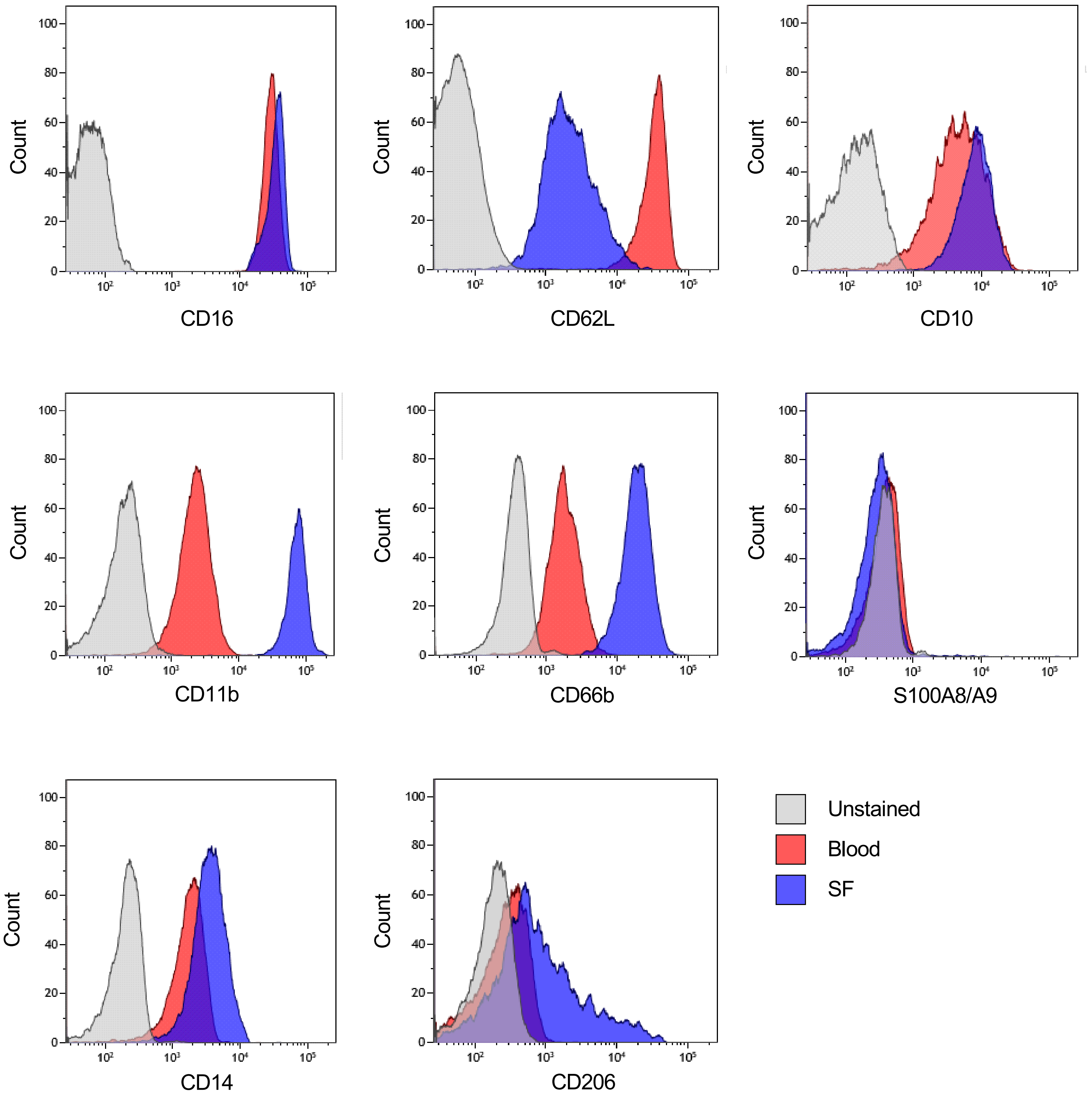

Supplemental Figure 2. Neutrophil surface markers.
Representative histograms of neutrophil surface marker stainings.

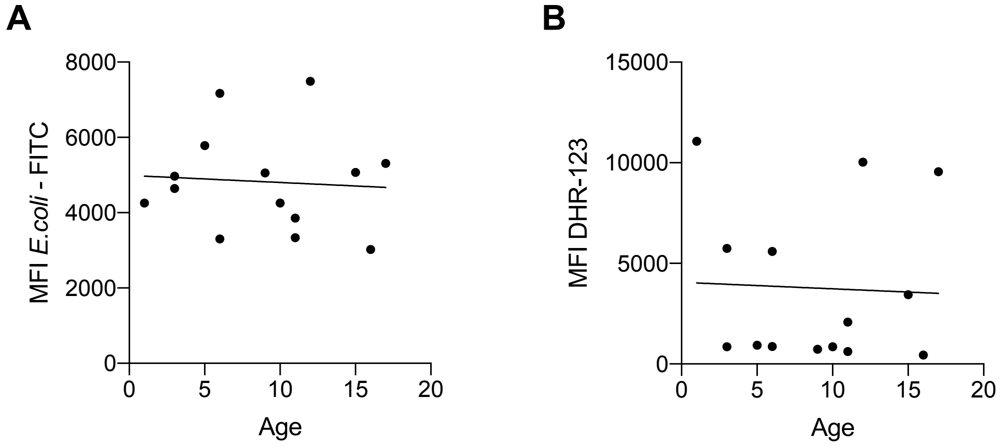

Supplemental Figure 3. Patient age does not influence neutrophil phagocytosis or oxidative burst.
Patient age in relation to phagocytosis (A) or ROS production (B).
